# Clinical measurement of tooth wear: Tooth wear indices

**DOI:** 10.4317/jced.50592

**Published:** 2012-02-01

**Authors:** Francisco J. López-Frías, Lizett Castellanos-Cosano, Jenifer Martín-González, José M. Llamas-Carreras, Juan J. Segura-Egea

**Affiliations:** 1 Associated Professor, Department of Endodontics, School of Dentistry, University of Sevilla; 2 Doctoral fellow, Department of Endodontics, School of Dentistry, University of Sevilla; 3 Associated Professor, Department of Orthodontics, School of Dentistry, University of Sevilla; 4 Professor, Department of Endodontics, School of Dentistry, University of Sevilla

## Abstract

Attrition, erosion, and abrasion result in alterations to the tooth and manifest as tooth wear. Each classification corresponds to a different process with specific clinical features. Classifications made so far have no accurate prevalence data because the indexes do not necessarily measure a specific etiology, or because the study populations can be diverse in age and characteristics. Tooth wears (attrition, erosion and abrasion) is perceived internationally as a growing problem. However, the interpretation and comparison of clinical and epidemiological studies, it is increasingly difficult because of differences in terminology and the large number of indicators/indices that have been developed for the diagnosis, classification and monitoring of the loss of dental hard tissue. These indices have been designed to identify increasing severity and are usually numerical, none have universal acceptance, complicating the evaluation of the true increase in prevalence reported. This article considers the ideal requirements for an erosion index. A literature review is conducted with the aim of analyzing the evolution of the indices used today and discuss whether they meet the clinical needs and research in dentistry.

** Key words:**Tooth wear, tooth wear indices, attrition, erosion, abrasion, abfraction.

## Introduction

**Figure 1:**
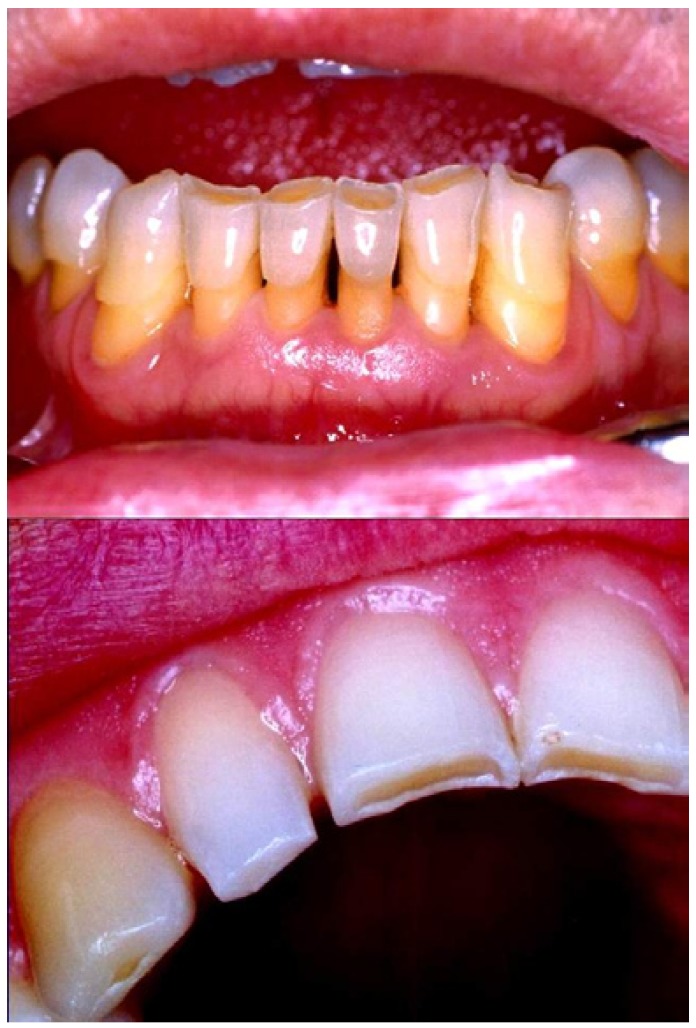
Attrition: loss of enamel, dentin, or restoration by tooth-to-tooth contact.

**Figure 2:**
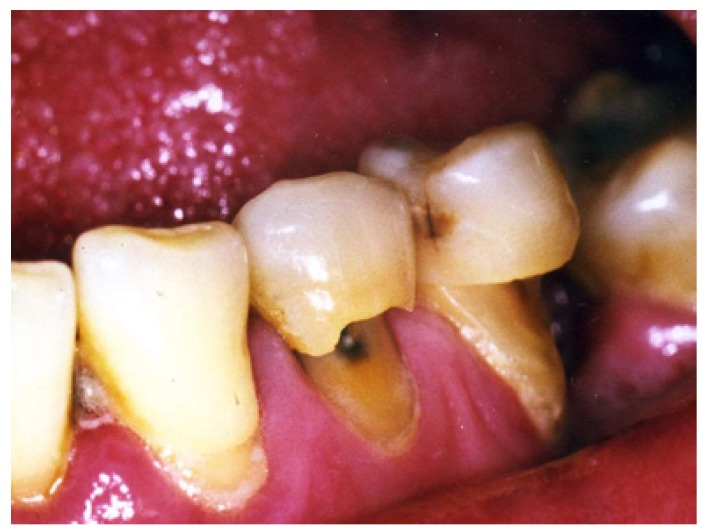
Abrasion: pathological wear of tooth substance through bio-mechanical frictional processes. These lesions are provoked by tooth brushing.

Tooth wear can be classified as attrition, erosion and abrasion. Attrition is defined as the loss of enamel, dentin, or restoration by tooth-to-tooth contact (1) (Fig. 1). Erosion is the loss of dental hard tissues by chemical action not involving bacteria (2). It is further classified, according to the source of the acid, as either intrinsic or extrinsic. Intrinsic sources of acids originate in the stomach and are associated with eating disorders, such as anorexia and bulimia nervosa (3), or with acid reflux and regurgitation (4). Extrinsic sources are acids contained in dietary components, such as carbonated soft drinks and fruit, and fruit juices (5,6). Abrasion is the loss of tooth substance from factors other than tooth contact (1) (Fig. 2). Perceptions relating to the relative importance of erosion, attrition, and abrasion are geographically polarized, with apparently lower recognition in North America of the potential consequences of acids in tooth wear. This apparent conflict arises from a differing interpretation of the definitions relating to the aetiology of tooth wear. Taking into account the increasing elderly population and that, nowadays, it is becoming less common to find elderly edentulous, tooth wear is a dental problem of maximum importance (6).

In 1908, Black (7) in his seminal work on Operative Dentistry discussed the problematic aetiology of what he termed ‘‘erosions’’ and stated that ‘‘Our information regarding erosion is far from complete and much time may elapse before its investigation will give satisfactory results’’. After considering each hypothesis in turn, finding fault with all, he concluded that he had no theory of his own to offer, which did not have features that rendered it impossible.Other researchers in the early part of the 20th century also considered these lesions(8).

In the last century many authors have described this type of injury, not being able to offer a reasonable explanation (9), in 1932 Kornfeld made the observation that in all cases of cervical erosion he noticed heavy wear facets on the articulating surfaces of the teeth involved and that the erosion tended to be at the opposite side of the tooth to the wear facet (10,11).

The confusing use of the term erosion to describe a lesion which may actually be caused by mechanical abrasion is further compounded by the fact that to a chemical engineer the process described by dentists as erosion is known as corrosion (12). This imprecise terminology has contributed both to the difficulty of carrying out good quality research and making accurate diagnoses, which would enable appropriate treatments to be recommended.

Many practitioners felt that over enthusiastic tooth- brushing and the use of abrasive toothpastes were the primary cause of these lesions but Lee and Eakle (13) put forward the hypothesis that tensile stresses created in the tooth during occlusal loading may have a role in the aetiology of cervical erosive lesions. They described three types of stress placed on teeth during mastication and parafunction: a) Compressive: the resistance to compression; b) Tensile: the resistance to stretching; and c) Shearing: the resistance to twisting or sliding.

The authors stated that in a ‘‘non-ideal’’ occlusion large lateral forces could be created which would result in compressive stresses on the side of the tooth being loaded and tensile stresses in the opposite side. As it was well known that enamel is strong in compression but weak in tension, it was suggested that those areas in tension were prone to failure. The region of greatest stress is found at the fulcrum of the tooth. The characteristic lesion described was wedge-shaped with sharp line angles and situated at or near the fulcrum of the tooth, where the greatest stress is generated. It was suggested that the direction of the lateral force governed the position of the lesion and its size was related to the magnitude and duration of the force.

Grippo put forward a new classification of hard tissue lesions of teeth (14). He defined four categories of tooth wear:

● Attrition: the loss of tooth substance as a result of tooth to tooth contact during normal or parafunctional masticator activity.

● Abrasion: the pathological wear of tooth substance through bio-mechanical frictional processes, e.g. tooth brushing.

● Erosion: the loss of tooth substance by acid dissolution of either an intrinsic or extrinsic origin, e.g. gastric acid or dietary acids.

**Figure 3:**
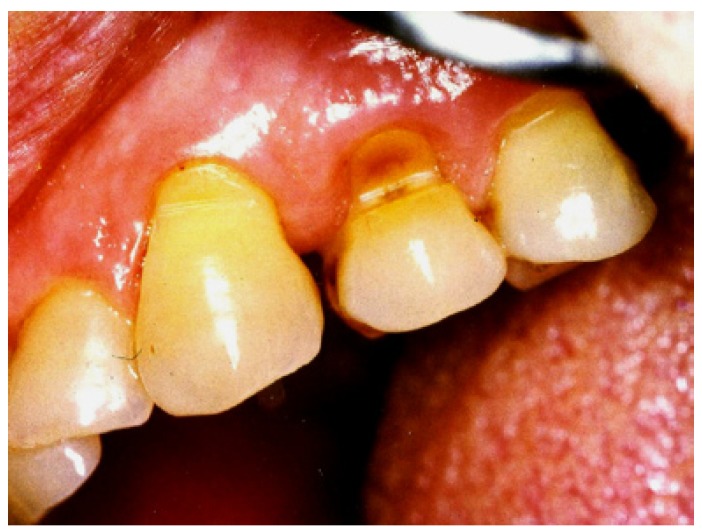
Abfraction: caused by flexure of the tooth during loading.

● Abfraction: the pathologic loss of tooth substance caused by bio-mechanical loading forces (Fig. 3). It was postulated that these lesions were caused by flexure of the tooth during loading leading to fatigue of the enamel and dentine at alocation away from the point of loading. The word ‘‘abfraction’’ was derived from the Latin ‘‘to break away’’.

Grippo then went on to further describe five categories of abfraction: hairline cracks, striations (horizontal bands of enamel breakdown), saucer-shaped (a lesion entirely within enamel), semi-lunar-shaped (a crescent-shaped lesion entirely within enamel), and cusp tip invagination (a depression on the cusp tip seen in molar and premolar teeth).

Lambert and Lindenmuth (15) considered that the profession should now consider occlusal stress as a primary factor in the creation of cervical notch lesions and a considerable body of theoretical work was accumulating to support the theory (15). To date it would appear that practitioners widely accept that abfraction is related to atypical occlusal loading despite there being a paucity of evidence other than purely theoretical to support this hypothesis.

## Clinical measurement of tooth wear

There is both a clinical and scientific need to be able to measure tooth wear, and the literature abounds with many methods which can be broadly divided into quantitative and qualitative in nature. Quantitative methods tend to rely on objective physical measurements, such as depth of groove, area of facet or height of crown. Qualitative methods, which rely on clinical descriptions, can be more subjective if appropriate training and calibration are not carried out but which, with correct safeguards, can be valuable epidemiological tools. In a clinical intraoral examination, there will be an inclination towards descriptive assessment measures, such as mild, moderate or severe, rather than quantitative measurement, which is easier to perform reliably on a model or in the laboratory. Such methods tend to be more sensitive but do not lend themselves readily to clinical use, especially in epidemiology, where fieldwork data collection is often carried out in an environment lacking sophisticated equipment.

Quantitative and qualitative methods typically utilise grading or scoring systems designed to identify increasing severity or progression of a condition; these are described as indices and are usually numerical. An ideal index should be simple to understand and use, clear in its scoring criteria and be demonstrably reproducible. Its application should be useful for research into the aetiology, prevention and monitoring of a condition, essentially being an epidemiological and clinical tool.

Review of the literature reveals the fact that many different tooth wear indices have been developed for clinical and laboratory use all over the world. Unfortunately, the production of so many indices does not allow for ready comparison of results between different working groups, and this is especially important in epidemiology when trying to define the prevalence of a condition. Confusion is further generated as the majority of researchers, in their attempts to quantify the amount of tooth tissue loss due to tooth wear, have historically concentrated only on one aetiology, and these indices tend to be surface limited.

Often, the wear patterns described do not appear to reflect the aetiology suggested, and this relates to lack of uniformity with tooth wear terminology and translation errors. Many diagnostic indices do not properly reflect the morphological defects, and there is little international standardisation. All of these factors complicate the comparison of data and evaluation of the efficacy of preventive and therapeutic measures.

The literature identifies different indices for use in clinical and laboratory situations and specific indices for attrition, abrasion, erosion and multifactorial tooth wear. There are common threads to all of the indices, such as descriptive diagnostic criteria and criteria for quantifying the amount of hard tissue loss. These generally consider the size of the affected area (as a proportion of a sound surface and/or the depth of tissue loss) often expressed as a degree of dentine exposure.

One area of consensus is the recognition of dentin exposure as an indicator for substantial loss of tooth tissue. It is a convenient cut off, and if applied leads to a dichotomous wear scoring system. Nonetheless, exposure of dentin is a dramatic finding in permanent teeth at young age.

## First tooth wear indices

It is perhaps significant that the earliest index documented by Broca was used as a foundation for the development of further indices graded horizontal or oblique patterns of occlusal wear without presupposing the aetiology. Smith and Knight (16) introduced the more general concept of measuring tooth wear per se, irrespective of the cause, and since then more recent indices have been developed or modified from Smith and Knight that do not rely on a prior diagnosis and are more clinically relevant. Most of these stress the importance of user training sessions and calibration exercises.

Smith and Knight (16) took Eccles’ ideas a stage further, producing the tooth wear index (TWI), a comprehensive system whereby all four visible surfaces (buccal, cervical, lingual and occlusalincisal) of all teeth present are scored for wear, irrespective of how it occurred ( Table 1). This avoids the confusion associated with terminology and translation or differences in opinion for diagnosis of aetiology based on clinical findings. Guidelines for using the criteria were produced in a booklet by the authors to aid training and standardisation with other investigators; in cases of doubt, the lowest score is given. Complete enamel loss (score 4) may, however, be misleading, as there is almost always a rim of enamel at the worn surface margins (the colloquial “enamel halo”).

**Table 1:**
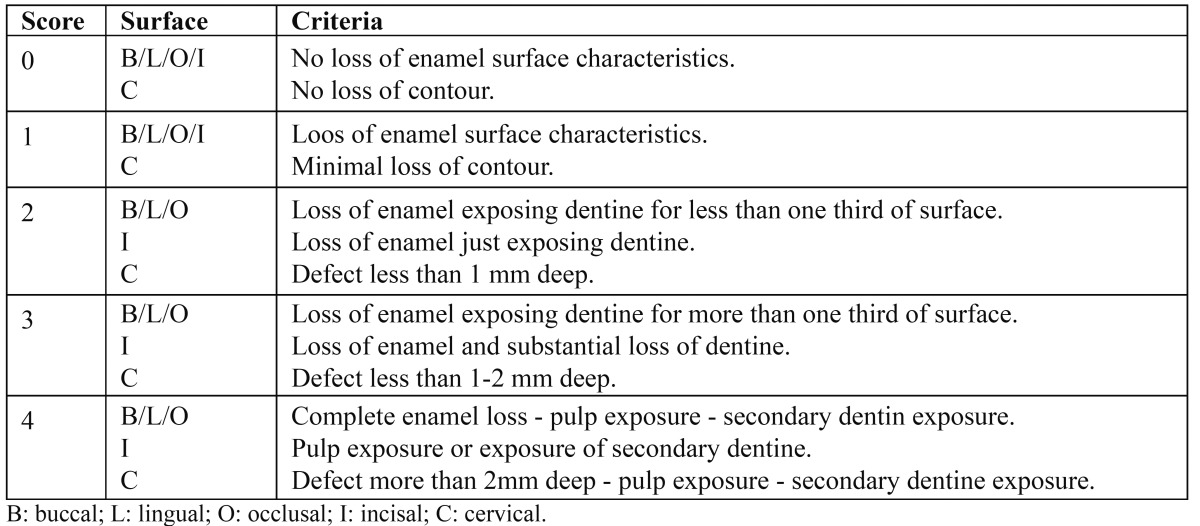
Smith and Knight tooth wear index (16).

This index was the first one designed to measure and monitor multifactorial tooth wear; a further pioneering feature was the ability to distinguish acceptable and pathological levels of wear. However, some problems have been identified with the TWI, including the time necessary to apply to a whole dentition, amount of data generated and the comparisons with threshold levels for each age group; the thresholds proposed were high, erring towards understatement rather than exaggerations of pathological wear. Full use of the index as a research tool is not feasible without computer assistance.

Over the past 20 years, there have been a number of studies reporting the prevalence of tooth wear. A recent systematic review from Kreulen et al. on tooth wear in adults showed that prevalence of severe tooth wear increases with age (17,18).

A special interest is the clinical measurement of erosion due to their prevalence in children and adolescents.The earliest indices shared common, arbitrary criteria, relying on descriptive terms such as slight, mild, moderate, severe and extensive. Restarski et al. (19) developed a six point grading system to evaluate the severity of erosive destruction observed on the lingual surfaces of rat and puppy molars, but concerns were raised with regards to reproducibility. With vague criteria definitions, variability in recording is expected. Each animal was allocated a total score, calculated by summing the mean molar quadrant scores. Whilst producing simple data for analysis, it is acknowledged that averaging scores in this manner leads to the loss of much data. If the number of teeth severely affected is small, the erosion score will be low; but this could mask a significant, localised clinical problem (20).

Eccles (21) originally classified lesions broadly as early, small and advanced, with no strict criteria definitions, thus allowing wide interpretation. Later, the index was refined and expanded, with greater emphasis on the descriptive criteria. It was presented as a comprehensive qualitative index, grading both severity and site of erosion due to non-industrial causes, and is considered as one of the cardinal indices from which others have evolved. In essence, it breaks down into three classes of erosion, denoting the type of lesion, assigned to four surfaces, representing the surface where erosion was detected ( Table 2).

**Table 2:**
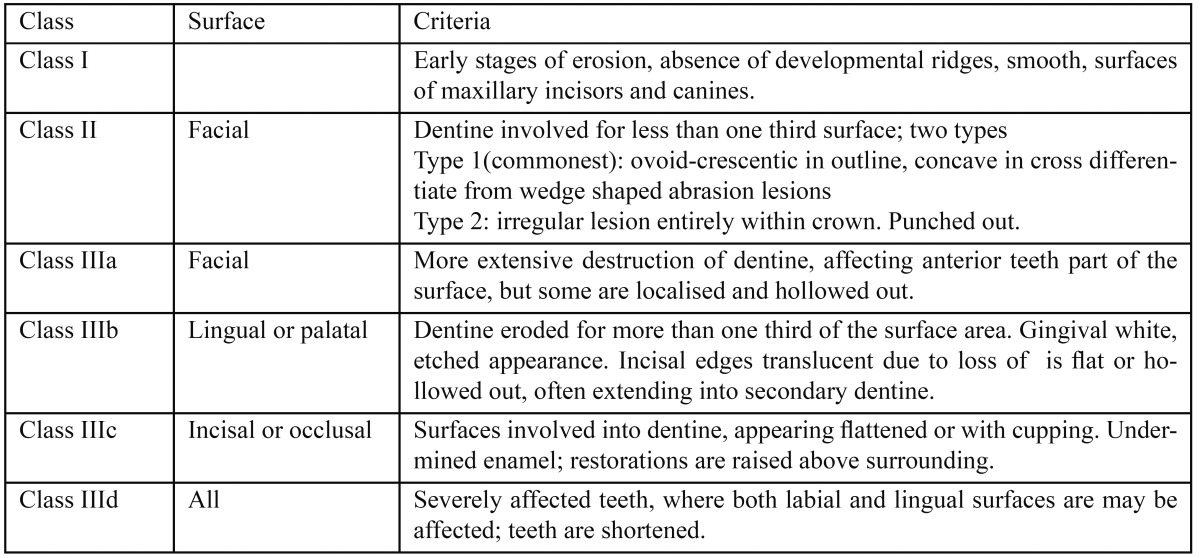
Eccles index for dental erosion of non-industrial origin (21).

Greater accuracy was introduced by Xhonga and Valdmanis (22) who divided erosions into four levels by measurement with a periodontal probe: none, minor (less than 2 mm), moderate (up to 3 mm) and severe (greater than 3 mm). They further differentiated types of erosion by morphological descriptions, such as wedge, saucer, groove and atypical. They did not address the problem of inter- or intra-examiner variability.

Developments of tooth wear indices

Many other indices have been proposed for measuring erosive tooth wear (23-26) which have their roots in the indices of Eccles (21) and Smith and Knight (16). Linkosalo and Markkanen (25) utilized a qualitative index with listed diagnostic criteria to confirm lesions as erosive and a four-scale grading of severity, relating to involvement of dentine.

Bardsley et al. (23) pioneered a new, simplified version of TWI (16) when carrying out epidemiological studies on large numbers of adolescents in North West England ( Table 3). Tooth wear scoring was essentially dichotomised into the presence or absence of dentine, with even cupping of dentine scoring one. A partial recording system was used, collecting data from 40 surfaces including occlusal surfaces of the four first molar teeth and the labial, incisal and lingual–palatal surfaces of the six upper and lower anterior teeth.

**Table 3:**
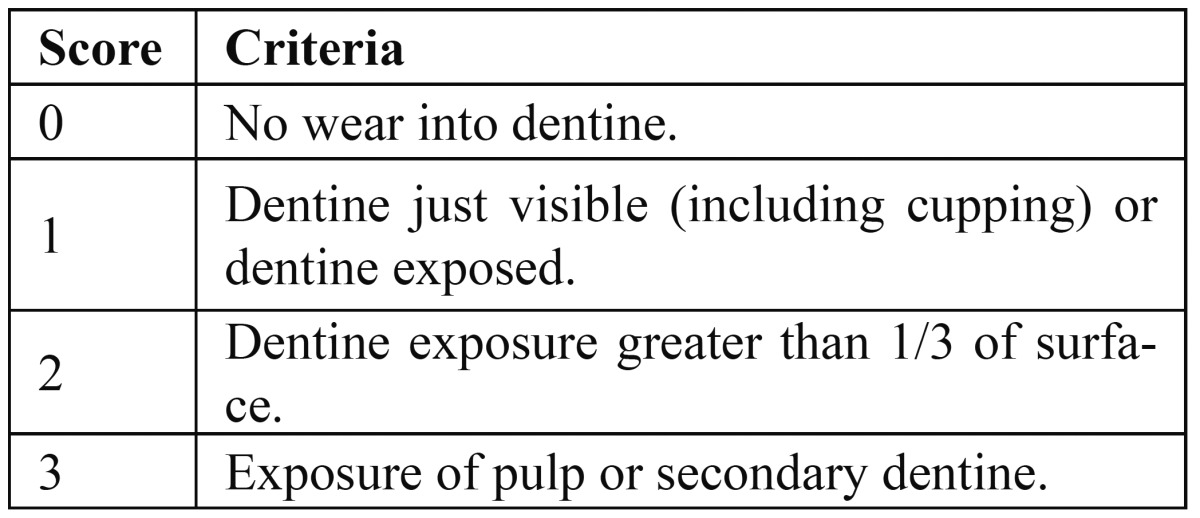
Simplified scoring criteria for tooth wear index (24).

However, despite calibration and training, difficulties were experienced diagnosing dentine exposure in the epidemiological field and there is some debate as to the significance of dentinal cupping when exposed dentine does not relate to significant amounts of tissue loss (28).

Oilo et al. (28) Concentrated on a different type of scoring system, with criteria based on treatment need. They criticized the use of indices that used a nonlinear scoring method, claiming calculated mean wear scores can be misleading.All groups were subdivided according to degree of dentine exposed and clinical findings such as pain, sensitivity and fracture of restorations, giving the impression of a cumbersome system. Dahl et al. (29) modified it with the introduction of even more categories, with an aim to establish subjective dental criteria for present and future evaluations of tooth wear and the need for treatment. In practice, these indices require experience for reliable use; individuals with differing clinical backgrounds will not get consistent, objective results.

Larsen et al. (8) recommended a new clinical index based on a combination of clinical examination, photographs and study casts, with complicated qualitative and quantitative criteria. Plaque-free teeth were clinically examined and photographed prior to taking silicone impressions for epoxy resin casts. They considered clinical and photographic data to be supplemental with final wear classification based on visual inspection of casts at ×10 magnifications.

There is agreement in scientific literature about the clinical diagnostic criteria for dental erosion, basically defined as cupping and grooving of the occlusal/incisal surfaces, shallow defects on smooth surfaces located coronal from the enamel-cementum junction with an intact cervical enamel rim and restorations rising above the adjacent tooth surface. This lesion characteristic was established from clinical experience and from observations in a small group of subjects with known exposure to acids rather than from systematic research (24).

## Conclusions

Review of the literature on indices for tooth wear is confusing; there are too many indices proposed and used, with lack of standardisation in terminology. There are many epidemiological studies reported, but it is difficult to quantify the increases in prevalence reported internationally, as results are not easily comparable. It is doubtful that any of the indices used is sensitive enough for all cases, also these can not be used to measure the wear rate. Is a challenge to try to develop a simple index that can be used clinically to assess progression of wear. To date, there is not one ideal index that can be used for epidemiological prevalence studies, clinical staging and monitoring, and it may be necessary to accept that one simple index does not yet exist to meet all requirements of both clinical and research teams. However, there should be an aim for indices that can be relevant to both fields and can be used internationally in order to strengthen knowledge of dental wear. Knowledge about the validity of current diagnostic criteria of different forms of tooth wear is incomplete, therefore further research is needed.
